# ASSESSMENT OF THE ACCURACY OF REAL-TIME CONTINUOUS GLUCOSE MONITORING AND ITS CORRELATED FACTORS IN CRITICALLY ILL PATIENTS

**DOI:** 10.1186/2197-425X-3-S1-A192

**Published:** 2015-10-01

**Authors:** MZ Lu, Y Kang

**Affiliations:** West China Hospital, Sichuan University, Chengdu City, China; West China Hospital, Sichuan University, Intensive Care Unit, Chengdu, China

## Introduction

Real-time continuous glucose monitoring system (RTCGMS) measures the interstitial glucose continuously,it displays the trend of glucose all day and it can detect the hypeglycemia and hypoglycemia fastly,reducing the glucose variability.Many studies have evaluated the accuracy of RTCGMS in the critically ill patients,but they have different opinions.

## Objective

To assess the accuracy of RTCGMS in critically ill patients and its correlated factors.

## Methods

From July 2014 to January 2015,22 patients were enrolled.All the subjects underwent RTCGMS(San MediTech) for five days. Capilllary glucose values through self-monitoring of blood glucose(SMBG)were input twice a day for adjustment. Meanwhile, arterial cannula was placed for arterial blood sampling. The value of RTCGMS,SGMB and arterial blood glucose(ABG) at the same time were recorded every 4 hours. Whether use vasoactive agents, glucocorticoids, continuous renal replacement therapy(CRRT),enteral nutrition(EN), parenteral nutrition(PN) or not were also recorded at the same point. The correlation of RTCGMS and SMBG, RTCGMS and ABG were analyzed by correlation analysis. The consistency was analyzed by Clarke error grid. Relative absolute difference(RAD) was used to assess the accuracy of RTCGMS, its correlated factors were also analyzed.

## Results

A total of 504 pairs of RTCGMS and SMBG[11.0(9.0-13.1)mmol/L vs.(11.3 ± 3.2)mmol/L, P < 0.05)],RTCGMS and ABG[11.0(9.0-13.1)mmol/L vs.(11.11 ± 3.32)mmol/L, P < 0.05] were collected.

① Spearman correlation analysis denoted that RTCGMS was both positively correlated with SMBG value (r = 0.820, P < 0.001) and ABG value ( r = 0.792, P < 0.001).

② Clarke error grid showed that 98.4% of paired RTCGMS-SMBG value located in Zone A and B;98.2% of paired RTCGMS-ABG located in Zone A and B.

③ The ovrerall RAD of RTCGMS-SMBG was 9.8%(4.2%-17.9%), the ovrerall median RAD of RTCGMS-ABG was 12.0%(6.1%-21.2%).

④ The median RAD of RTCGMS-SMBG: in patients use vasoactive agents or not were 9.4% vs.10.3% (P > 0.05);in patients use glucocorticoids or not were 10.9%vs.9.7% (P > 0.05);in patients underwent CRRT or not were 10.0% vs.9.7% (P > 0.05);in patients used EN or not were 9.4% vs.10.3% (P > 0.05);in patients used PN or not were 10.0% vs.9.7% (P > 0.05); The median RAD of RTCGMS-ABG: in patines use vasoactive agents or not were 11.7% vs.12.6% (P > 0.05);in patients use glucocorticoids or not were 13.1%vs.11.8% (P > 0.05);in patients underwent CRRT or not were 13.2% vs.11.3% (P > 0.05);in patients used EN or not were 11.7% vs.12.6% (P > 0.05);in patients used PN or not were 13.2% vs.11.3% (P > 0.05)

## Conclusions

① The results of RTCGMS in critically ill patients are accurate,and have good correlation with SMBG and ABG.

② Conventional operations or therapies in ICU may not influenced the accuracy of RTCGMS.

## Grant Acknowledgment

Supported by grants from San MediTech Medical science and Technology Co., Ltd.
